# A Retrospective Study of the Prognostic Patterns in Colorectal Cancer Patients

**DOI:** 10.7759/cureus.38522

**Published:** 2023-05-04

**Authors:** Jayaditya Devpal Patil, Yusuf Mahdi Mohamed, Raed AlMarzooq

**Affiliations:** 1 Obstetrics and Gynaecology, University Hospitals of Leicester NHS Trust, Leicester, GBR; 2 Emergency Medicine, Salmaniya Medical Complex, Manama, BHR; 3 General Surgery, Al Kindi Hospital, Manama, BHR

**Keywords:** tumor stage, tumor grade, prognosis, screening, colorectal cancer

## Abstract

Introduction

Colorectal cancer (CRC) management has advanced globally, leading to a steady decline in mortality rates. However, recent studies have shown that the prognosis of CRC varies based on the anatomical site of the primary tumor, histopathological grading, and type of mutation. With an increase in the incidence of CRC globally and in Bahrain, there is a need for a recent descriptive study to improve overall management. This study aims to investigate the anatomical, histopathological, and molecular prognostic factors in CRC patients presenting to the Salmaniya Medical Complex (SMC).

Methods

The study was conducted retrospectively using ISEHA electronic database over two years (January 2019 to December 2020). A total of 101 patients with primary CRC registered in the General Surgery Department were included in this study. The sample size was further stratified and analyzed using descriptive statistics based on the available data of measured outcomes.

Results

Anatomical data showed that 65% of CRC patients had a tumor on the left side of the colon, 27.7% on the right side, and 7% in the transverse colon. Overall, 16.8% of all patients had rectal involvement. Histopathological data showed that 86% of the patients had a low-grade CRC adenocarcinoma. The most diagnosed tumor stage was pT3N0M0 (22.8%). In addition, there were ten metastatic cases (10 to the liver, of which three had concomitant lung involvement and two had concurrent brain metastases). The average tumor diameter was 46.2 mm, where 63% ranged between 30 mm to 69 mm. Most mutations involved the *TP53 *(27.7%) and the *KRAS *(29%) genes.

Conclusion

The study found that majority of CRC patients at SMC in Bahrain had relatively good overall anatomical, tumor staging and grading prognostic factors but somewhat poorer molecular prognostics.

## Introduction

Colorectal cancer (CRC) is the third most common cancer worldwide, with a global incidence rate of 6.1% and a mortality rate of 9.2%. In 2018 alone, there were 1.09 million new cases and 551,000 deaths secondary to CRC [1]. A retrospective study conducted to evaluate the epidemiology of cancers in Bahrain between 1998 and 2011 found CRC to account for 10.2% of all cancers in males. The study also reported a continual rise of CRC incidence over the years. Age-specific incidence among males was prominent in the age group of 25 to 29 years and steadily increased to peak in the age group of 70 to 74 years [2]. The incidence of CRC has also been increasing in other Middle Eastern and North African countries, including Bahrain [3]. Over the years, there have been numerous advances in CRC treatment leading to a steady decline in mortality. Most cases are often treated by surgical resection; however, the prognosis differs in terms of the anatomical site, type of mutation, and tumor stage [4,5]. Primary tumors can arise from different anatomical locations in the large bowel. Clinically, CRC can be classified as either right-sided CRC (RCRC) or left-sided CRC (LCRC). Understanding the presenting patterns of primary CRC based on the anatomical location is beneficial for management as LCRC responds better to adjuvant chemotherapy than RCRC [6]. Several studies have even suggested that the anatomical location of the tumor can also influence disease progression. RCRC tends to have poorer histological grading, staging, and even prognosis compared to primary tumors arising from the left side. The reasons for these differences are quite complex, but theories suggest a combination of several variables such as genetics, embryological origin, histology, and anatomical location to play a role [7-9]. Molecular mutations have often been implicated in the pathogenesis and overall prognosis of CRC. Several studies have shown that patients with specific genetic mutations, particularly in the KRAS gene, often have poorer prognosis and reduced overall survival rate compared to patients with TP53 or APC tumor suppressor gene mutations. It is a lot more challenging to medically treat CRC patients with multiple genetic mutations [10]. Currently, limited literature has been published on the distribution of CRC at local population levels, and, to our knowledge, no study on the prognostic factors in particular has been reported in Bahrain. The primary outcome of this study is to investigate the different molecular, anatomical, and histopathological prognostic factors in primary CRC patients presenting to the General Surgery Department at the Salmaniya Medical Complex (SMC) hospital in Bahrain. The secondary outcomes will investigate the gender, nationality, and age demographics of CRC patients. This study provides valuable data on CRC distribution in Bahrain and will allow for improved overall treatment, guide formation of management algorithms, improve patient satisfaction, and serve as a basis for future studies in the field. To our knowledge, there is no current widely established screening program for CRC in Bahrain; therefore, this study can potentially aid in determining the cut-off age for CRC screening.

## Materials and methods

This study was conducted retrospectively by reviewing available data from the ISEHA electronic database in SMC. A patient list was generated by containing data over a two-year period from January 2019 to December 2020 from the General Surgery Department. Patients diagnosed with primary CRC at the General Surgery Department in SMC during the study period were included, regardless of age, gender, or nationality. However, patients diagnosed at other medical institutes prior to referral to SMC did not have any available records for collection and were excluded. Patients diagnosed with secondary CRC, polyps, and benign lesions were also excluded from the study. The medical and histopathological records were retrieved from the electronic database. Demographic information such as age, gender, nationality, and comorbidities was obtained from medical history reports. Information on the anatomical tumor location, tumor size, and tumor stage was obtained from pathology reports. Data on grade and type of malignancy were reviewed from histology reports. Genetic laboratory reports were also accessed to retrieve all available information on genetic mutations. No human or animal trials were undertaken. All data relevant to measuring the primary and secondary outcomes were collected and recorded on a Microsoft Excel sheet. No patient identifiers were used, and all data were encrypted and stored on the research team’s computer system with access available only to the research team. The collected data were analyzed using simple descriptive analysis. Issues with data tracking in the medical database were encountered, particularly for genetic laboratory reports where data was missing and inconsistent.

## Results

A total of 101 patients with primary CRC presenting to SMC’s General Surgery Department during the two-year study period were included in our study. Data on tumor size was available for 94 patients, and 55 patients had available data on underlying genetic mutations. All 101 patients had available data on anatomical tumor location, tumor stage, histopathological grade, and demographic information. Demographic data comprising of age, gender, comorbidities, and nationality of all patients are described in Table 1. With regard to gender and age, male patients accounted for 60.4% of all CRC cases (male-to-female ratio: 1.53:1), and the average age was 56 years, where 75.2% of patients were older than 50 years. With regard to patient nationality, around two-thirds of the patients were Bahraini nationals, while the rest were non-Bahraini. Data on the specific ethnicity of the non-Bahraini patient population and family history were unavailable. Associated comorbidities were seen in 53% of patients. Hypertension (HTN), type 2 diabetes mellitus (DM2), hyperlipidemia, and obesity were very common and seen in varying combinations among patients. The combination of DM2 and HTN was seen in 6%, and HTN, DM, and hyperlipidemia was seen in 7% of patients. Only two patients had a history of inflammatory bowel disease.

**Table 1:** Demographic and clinical characteristics of CRC patients CRC, colorectal cancer

Demographic variables		Number of CRC patients						
		Both genders		Male		Female		
Age group								
20-29		1		1		-		
30-39		11		6		5		
40-49		13		11		2		
50-59		27		12		15		
60-69		35		20		15		
70-79		14		11		3		
Total (%)				61 (60.4%)		40 (39.6%)		
Mean (x̅)		56 (SD 11.82)						
Male-to-female ratio				1.53 : 1				
Nationality		Both genders		Male		Female		
		N	%					
Bahraini	Yes	63	62.38%	36		27		
	No	38	37.62%	25		13		
Number of chronic comorbidities		Both genders		Male		Female		
		N	%	N	%	N	%	
None		47	46.53%	23	37.7%	24	60%	
One		20	19.80%	16	26.23%	4	10%	
Two		14	13.86%	11	18.03%	3	7.50%	
Three		14	13.86%	7	11.47%	7	17.50%	
More than three		6	5.94%	4	6.55%	2	5%	

The anatomical distribution is illustrated in Table 2. Results showed that the sigmoid colon was involved in 32.7% of the cases, while the rectum was involved in 16.83%. The splenic flexure was the least involved segment of the bowel.

**Table 2:** Distribution of tumors according to anatomical location *Transverse colon is an exception, since two-thirds of the colon originated embryologically from the midgut, while one-third originated from the hindgut, making it difficult to classify as solely part of RCRC or LCRC.

Anatomical location	Frequency (%)
Right-sided primary tumors	
Cecum (Ileocecal included)	14 (50.00%)
Ascending colon	10 (35.71%)
Cecum and ascending colon	1 (3.57%)
Cecum, ascending colon, and transverse colon	1 (3.57%)
Hepatic flexure	2 (7.14%)
Total	28 (27.72%)
Left-sided primary tumors	
Splenic flexure	1 (1.51%)
Descending colon	6 (9.09%)
Sigmoid	33 (50%)
Descending colon and sigmoid	1 (1.51%)
Recto-sigmoid junction	8 (12.12%)
Rectum	17 (25.76%)
Total	66 (65.35%)
Primary tumor occurring in both sides of the colon*	
Transverse colon	7 (100%)

The pTNM eighth edition was used for tumor staging. The most commonly diagnosed pTNM stage was pT3N0M0 (22.8%). There were 10 cases of metastases, with the liver involved in all 10. The lungs were involved in three cases, while the brain showed evidence of metastases in only two cases. All cases of lung and brain metastases had concomitant liver metastases as well. Nodal involvement was reported in 57.4% cases. As shown in Table 3, 57.4% of the CRC patients had moderately differentiated adenocarcinoma. Only four cases were poorly differentiated tumors, and seven cases were moderate to poorly differentiated tumors. Five cases were diagnosed with mucinous adenocarcinoma variant, two cases had signet cell subtype, one case had a papillary variant, and one case had adenocarcinoma associated with necrosis. Four cases of adenocarcinoma were associated with ulceration. Three cases involved neuroendocrine colon cancer: two cases were moderately differentiated and only one case was found to be well-differentiated. As shown in Table 4, the average diameter of the tumor was about 46.16 mm. Approximately 63% of tumors ranged from 30 mm to 69 mm in size. The largest tumor diameter was 134 mm.

**Table 3:** Histopathological grading of the tumor in CRC patients CRC, colorectal cancer

Subtypes	Histopathological grading (number of CRC patients)			
	Well differentiated	Moderately differentiated	Moderately to poorly differentiated	Poorly differentiated
Adenocarcinoma	16	58	7	
Necrosis		1	1	
Ulcer		4		
Signet cell type		2		
Mucinous variant		2		
Papillary variant		1		
Mucinous, adenocarcinoma type				2
High-grade dysplasia				1
Neuroendocrine	1	2		
Mucinous carcinoma				1
Unknown				2
Total	17	70	8	6
Frequency	16.83%	69.31%	7.92%	5.94%

**Table 4:** Tumor diameter range in CRC patient *Missing data are not included in calculation CRC, colorectal cancer

Tumor size (mm)	Number of CRC patients	Frequency (%)
Missing data	7	6.93%
10-19 mm	3	2.97%
20-29 mm	9	8.91%
30-39 mm	32	31.68%
40-49 mm	11	10.89%
50-59 mm	9	8.91%
60-69 mm	12	11.88%
70-79 mm	7	6.93%
80-89 mm	8	7.92%
120-129 mm	2	1.98%
130-139 mm	1	1.00%
Total	101	
Average (mm)*	46.16	

As shown in Table 5, the KRAS (29%) and TP53 (27.7%) genes accounted for majority of the mutations in our patients.

**Table 5:** Characteristics of genetic mutation in CRC patients CRC, colorectal cancer; MS, microsatellite

Genetic variables		N	%
Has mutated gene?	Yes	55	54.56%
	No	46	45.54%
Type of mutated gene	Frequency of patients with accompanying genetic mutation		
CDX2	6 (10.91%)		
BRAF	2 (3.64%)		
TP53	15 (27.72%)		
CK20	2 (3.64%)		
CK7	1 (1.82%)		
KRAS (wild type)	16 (29.09%)		
Resistant EGFR-KRAS	1 (1.82%)		
MSH2	5 (9.09%)		
MSH6	5 (9.09%)		
MLH1	5 (9.09%)		
PMS2	6 (10.91%)		
Synaptophysin	3 (5.54%)		
Chromogranin	2 (3.64%)		
NRAS	1 (1.82%)		
MS instability	1 (1.82%)		

## Discussion

Analysis of our data reported a male-to-female ratio of 1.53:1. This supports the established distribution of CRC to be commoner in men [11]. Mortality rates are significantly higher in men than women of all age groups, with the widest gap at the ages of 70-74 years [12]. These results support a slightly poorer prognosis. The youngest patient with primary CRC in our study was 27 years old. Although young and well below the average age group, one study concluded a younger age (<35 years) was not considered an independent risk factor for prognosis [13]. Many studies have also shown young adults with CRC to have similar outcomes as old patients [14]. Our results found 75% of the patients to be between 50 and 79 years of age, and the mean age to be 56 years, supporting established data on age distribution of CRC. These data also offer the potential to establish a cut-off age for CRC screening programs in Bahrain. With regard to associated comorbidities, DM2, HTN, and hyperlipidemias were seen in multiple patients, highlighting the association between obesity, DM2, and increased risk of CRC [15]. Interestingly, some studies have shown an inverse association between serum cholesterol levels and incidence of CRC [16]. With this in mind, the 20 patients with associated hyperlipidemia in our study may potentially have better prognosis. One study highlighted a similar association in Hawaiian Japanese men; however, these results were confined to a specific cohort and may not be replicable to the population in Bahrain [17]. Inflammatory bowel disease was seen in two patients and is a known risk factor and indicator for poor prognosis [18]. Certain postoperative complications such as infection and sepsis have been shown to be increased in patients with BMI ≥ 30 kg/m2, ASA score > 2, and patients over 70 years, along with chronic steroid use. Overall, these complications are associated with increased morbidity, extended postoperative stay, readmission, and increased financial burden, leading to a poorer prognosis [19]. Our study found that almost two-thirds of the patients had tumor in the left side of the colon and only seven patients had involvement of the transverse colon. This highlights that most of the patients hold better prognosis as LCRC has been reported to respond better to adjuvant chemotherapy treatment compared to RCRC, which tends to have poorer histological grade, stage, and prognosis [6,7]. In terms of histopathology, 86.1% of the patients had a low-grade CRC adenocarcinoma (well and well to moderately differentiated), a good prognostic factor. Two patients had the mucinous adenocarcinoma variant, which holds poor prognosis [20]. Two patients were diagnosed with signet cell adenocarcinoma, an independent predictor of poor survival [21]. Only two cases of adenocarcinoma were associated with necrosis. Tumor necrosis is associated with the worst prognosis and tends to be resistant to chemo and radiotherapy [22]. One case was of a papillary adenocarcinoma variant, which also has poor prognosis due to higher frequency of infiltration, deeper bowel wall penetration, and increased probability of positive lymph nodes compared to conventional adenocarcinomas [23]. Although these cases have a relatively poor prognosis, they account for only a small portion of the sample size. In terms of tumor stage, the pTNM classification system offers a far superior and precise definition of primary tumor invasion and lymph node involvement compared to Duke’s criteria, which only assess tumor invasion. Another disadvantage of Duke’s criteria is that varying outcomes are observed in patients within each stage [24]. Results showed that most of our patients staged pT3N0M0 (22.8%), pT4a/bN2a/bM0 (17.8%), and pT2N0M0 (13.9%). There were 10 cases of metastases, and these cases had a tumor stage of pT3N0M1 and pT4N1M1, which suggests that the overall tumor staging pattern in the patients at SMC seemed to support a good prognosis. Positive lymph nodes were reported in 58 cases. Studies have shown that lymph node yields may serve as prognostic indicators [25]. Although more than half of our patients reported positive lymph nodes, further details on lymph node ratio and location of lymph node retrieval were not available during data collection. This deficit in data makes it difficult to predict the prognosis for our patients with regard to lymph node involvement. Another significant histological finding from our study was the average tumor diameter. During data retrieval, 94 patients had available data on tumor diameter, and the average diameter was 46.16 mm. Studies have shown the maximum horizontal tumor diameter to be a variable in determining prognosis [26]. A tumor diameter of more than 45 mm in CRC patients has slightly worse five-year survival rates; however, these outcomes also depend on the tumor location. From the study population, the average tumor diameter is close to the baseline average reported in several other studies looking at similar prognostic factors [27]. Larger tumor diameters are associated with poor prognosis, particularly the case with a 134-mm diameter. Although the average diameter was more than 45 mm, most tumors were associated with low-to-moderate grade and involved the left colon. Accurately concluding the prognosis using the maximum tumor diameter may be challenging as this involves a complex interaction between tumor grade and histology. Tumor stage may also potentially affect the accuracy of tumor size measurements, where maximum tumor diameter may not reflect the extent of bowel wall infiltration [28]. In terms of molecular and immunohistochemical findings, TP53 gene and KRAS gene mutations were involved in most cases. KRAS mutations accounted for 29% of the cases, which is interesting as mutant KRAS has been considered as a poor prognostic factor; however, some studies have debated its role as a prognostic marker [29,30]. One patient with mutated KRAS was also noted to have EGFR (epidermal growth factor receptor) treatment resistance, leading to an even worse prognosis. TP53 gene mutations, seen in 27.7% of patients, have been associated with slightly increased malignant potential and chemo-resistance [31]. These primary outcomes could possibly be attributed to most of the patients having LCRC compared to RCRC, as several studies found LCRC to have better histopathological and molecular findings compared to RCRC [6,7]. Some notable studies have highlighted a significant association between the type of genetic mutation involved and the anatomical location of the bowel in cases of CRC. In one retrospective large-sampled survey by Loree et al., RAS, MSI, and BRAF gene mutations occurred at higher incidence rate on the anatomical right side of the colon than on the left [32]. Furthermore, in comparison, there was a higher rate of TP53 mutation incidence on the left side. Some mutations, such as the KRAS mutation, followed a unique anatomical pattern where it was noted more frequently on both the right side of the colon, and with the addition of the sigmoid and rectum regions of the left side of the colon. These results have shown practical importance, particularly in outlining the chemotherapy regimen in management of CRC. A meta-analysis study conducted by Holch et al. showed variable responses toward anti-EGFR therapy based on the genetic profile and anatomical location of metastatic CRC [33], with CRC mutations occurring on the right side having poorer response to anti-EGFR therapy in contrast to LCRC. Those with a wild-type RAS mutation of the LCRC had the greatest response in anti-EGFR therapy in contrast to anti-VEGF (vascular endothelial growth factor) therapy when added to standard management. However, it is a complex multifactorial topic that is under further research due to the heterogeneity of genetic profiles in CRC patients. Although our study's primary outcome and design were to collect local epidemiological data on various prognostic factors, it highlights the importance of continuous monitoring the genetic profile of CRC patients in Bahrain. Overall, the data collected was significant; however, this study had its limitations. Issues with data tracking were encountered throughout the study period as some data were not available or incomplete. Selection bias may have limited the validity of the results; however, a larger multi-center study in Bahrain may allow for more accurate results. We hope this study paves the way toward expanding the dataset of CRC cases to include all tertiary hospitals in the country, both public and privatized institutes.

## Conclusions

Majority of CRC patients presenting to SMC in Bahrain have better overall anatomical and histopathological prognostic factors but somewhat poor molecular prognostic factors. This study provides valuable data on CRC distribution in Bahrain and encourages further research in the field, allowing for a better understanding of CRC manifestation and its impact. A larger national multi-center study would provide a more comprehensive picture of the overall pattern and distribution of CRC in Bahrain. Demographic data will also be vital in establishing cut-off ages for screening programs in Bahrain.
